# Vasodilatory Effect of *Alpinia officinarum* Extract in Rat Mesenteric Arteries

**DOI:** 10.3390/molecules27092711

**Published:** 2022-04-22

**Authors:** Chae Eun Haam, Seonhee Byeon, Soo Jung Choi, Soyeon Lim, Soo-Kyoung Choi, Young-Ho Lee

**Affiliations:** 1 Department of Physiology, Yonsei University College of Medicine, Seoul 03722, Korea; cehaam94@gmail.com (C.E.H.); seonhee89@yuhs.ac (S.B.); 2 Department of Food and Biotechnology, Korea University, Seoul 02841, Korea; behindcsj@gmail.com; 3 Institute for Bio-Medical Convergence, College of Medicine, Catholic Kwandong University, Gangneung 25601, Korea; slim724@cku.ac.kr

**Keywords:** *Alpinia officinarum*, mesenteric resistance arteries, eucalyptol, vasodilation, relaxation, Ca^2+^

## Abstract

Background: *Alpinia officinarum* (*A. officinarum*) is known to exhibit a beneficial effect for anti-inflammatory, anti-oxidant, and anti-hyperlipidemic effects. However, no sufficient research data are available on the cardiovascular effect of *A. officinarum*. Thus, in this study, we investigate whether *A. officinarum* extract has direct effects on vascular reactivity. Methods: To examine whether *A. officinarum* extract affects vascular functionality, we measured isometric tension in rat mesenteric resistance arteries using a wire myograph. After arteries were pre-contracted with high-K^+^ (70 mM), phenylephrine (5 µM), or U46619 (1 µM), *A. officinarum* extract was treated. Results: *A. officinarum* extract induced vasodilation in a concentration-dependent manner, and this effect was endothelium independent. To further investigate the mechanism, we incubated arteries in a Ca^2+^-free and high-K^+^ solution, followed by the cumulative addition of CaCl_2_ (0.01–2.5 mM) with or without *A. officinarum* extract (30 µg/mL). Pre-treatment of *A. officinarum* extract reduced the contractile responses induced by cumulative administration of Ca^2+^, which suggests that extracellular Ca^2+^ influx was inhibited by the treatment of *A. officinarum* extract. These results were associated with a reduction in phosphorylated MLC_20_ in VSMCs treated with *A. officinarum* extract. Furthermore, eucalyptol, an active compound of *A. officinarum* extract, had a similar effect as *A. officinarum* extract, which causes vasodilation in mesenteric resistance arteries. Conclusion: *A. officinarum* extract and its active compound eucalyptol induce concentration-dependent vasodilation in mesenteric resistance arteries. These results suggest that administration of *A. officinarum* extract could exert beneficial effects to treat high blood pressure.

## 1. Introduction

Cardiovascular disease (CVD) remains the leading cause of deaths worldwide, responsible for 17.3 million deaths in 2018 [1]. Hypertension is known to be the major risk factor for CVD [2]. Thus, it is substantial to prevent and treat hypertension to reduce the risk of CVD. Regardless of the origin of the hypertension, elevated blood pressure is caused by either an increase in cardiac output or vascular resistance. The important proximal resistance arteries, such as mesenteric arteries, significantly contribute to the vascular resistance [3]. These small arteries that have a lumen diameter between 100–400 μm carry blood from the heart to various organs, according to their demand. The principal cause of elevated vascular resistance is a decrease in the lumen diameter of arteries [4]. On the contrary, arterial dilation leads to an immediate decrease in arterial blood pressure. Therefore, it is important to find effective vasodilatory substances to lower blood pressure. Although more than 200 antihypertensive drugs are available, there are limitations of low efficacy, side effects, and reduction in cardiovascular risk [5]. Recently, vasodilator compounds isolated from plants have been suggested as a new therapeutic target for preventing and treating hypertension [6].

*Alpinia officinarum* Hance (*A. officinarum*) is a plant that commonly grows in Asian countries [7]. The rhizomes of *A. officinarum* are widely used as a food additive and herbal medicine, for relieving colds, stomach aches, and swellings [8]. *A. officinarum* has also been used in European countries as a spice for over 1000 years [9]. Many studies have reported its pharmacological effects, such as anti-inflammatory [10], antioxidant [11], and antibacterial activities [12], as well as an anti-hyperlipidemic effect [13]. Although *A. officinarum* has been suggested to provoke beneficial effects, no sufficient research data are available on the cardiovascular effect of *A. officinarum*. Therefore, in the present study, we examined whether *A. officinarum* extract has a direct vascular effect, and, if so, what the underlying mechanism is.

## 2. Results

### 2.1. Effect of A. officinarum Extract on the High-K^+^- or Phenylephrine- or U-46619-Induced Contraction in Rat Mesenteric Arteries

**Figure 1:**
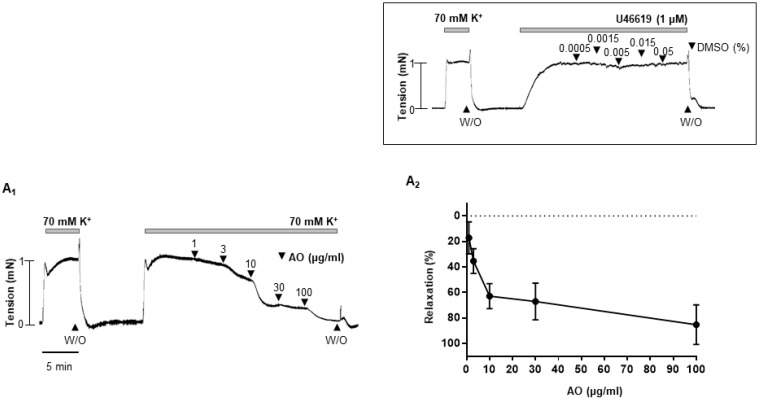

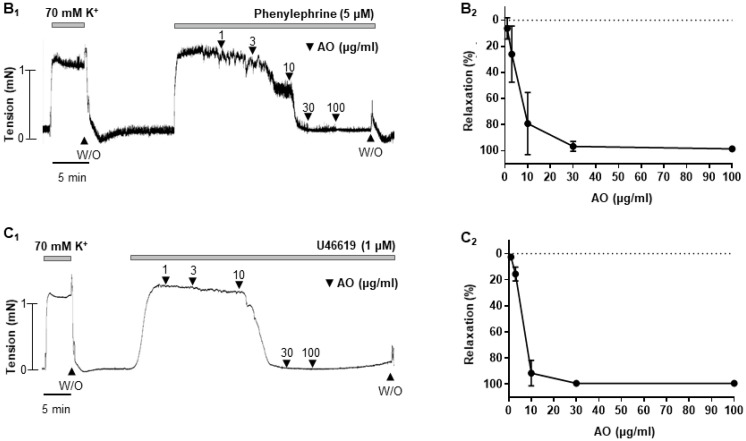
*A. officinarum* extract-induced vasodilation in rat mesenteric arteries. (**A_1_**–**C_1_**), data showing responses to cumulative administration of *A. officinarum* extract (1–100 μg/mL) on high-K^+^ (**A_1_**) or U46619 (**B_1_**) or phenylephrine (**C_1_**) -induced contraction. (**A_2_**–**C_2_**), statistical analysis of the relaxation response to *A. officinarum* extract. Mean ± SD (n = 7). Inset, representative trace showing responses to vehicle, DMSO (0.0005–0.05%). (W/O: wash out; AO: *Alpinia officinarum* extract).

*Alpinia officinarum* extract concentration-dependent dilated rat mesenteric arteries were pre-contracted with high-K^+^ solution (70 mM) or phenylephrine (5 μM) or U-46619 (thromboxane analogue, 1 μM) (Figure 1A-C). Regardless of the type of the vasoconstrictors, *A. officinarum* extract induced significant relaxation. The vehicle, dimethyl sulfoxide (DMSO, 0.0005–0.05%), had no significant effect on pre-contracted arteries with U-46619. (Figure 1 inset).

### 2.2. A. officinarum Extract Induced Endothelium-Independent Relaxation

**Figure 2:**
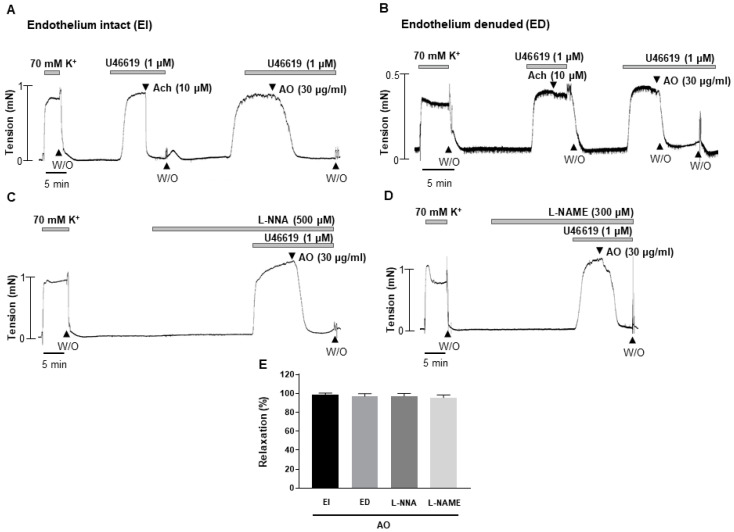
Endothelium-independent vasodilation induced by *A. officinarum* extract. (**A**), *A. officinarum* extract-induced vasodilation in the endothelium intact mesenteric arteries. (**B**), *A. officinarum* extract-induced vasodilation in the endothelium denuded mesenteric arteries. (**C**), *A. officinarum* extract-induced vasodilation in the presence of eNOS inhibitor L-NNA (500 μM). (**D**), *A. officinarum* extract-induced vasodilation in the presence of eNOS inhibitor L-NAME (300 μM). (**E**), statistical analysis of *A. officinarum* extract-induced vasodilation. Mean ± SD (n = 5). (Ach: acetylcholine; W/O: wash out; AO: *Alpinia officinarum* extract; L-NNA: N-ω-Nitro-L-arginine; L-NAME: N-ω-Nitro-L-arginine methyl ester).

To investigate the underlying mechanisms of *A. officinarum* extract-induced vasodilation, *A. officinarum* extract was applied in endothelium-intact (EI) or endothelium-denuded (ED) mesenteric arteries (Figure 2A,B). There was no significant difference between endothelium-intact or endothelium-denuded mesenteric arteries. To confirm the effect of *A. officinarum* extract on endothelium-dependent relaxation, arteries were pre-incubated with two endothelial nitric oxide synthase (eNOS) inhibitors, N-ω-Nitro-L-arginine (L-NNA, 500 μM) and N-ω-Nitro-L-arginine methyl ester (L-NAME, 300 μM), for 20 min, before being contracted with U46619 (1 μM, Figure 2C,D). The L-NNA and L-NAME did not affect the *A. officinarum* extract-induced relaxation, indicating that the vasodilatory effect of *A. officinarum* extract was not related with eNOS. This result suggests that *A. officinarum* extract-induced relaxation is endothelium-independent.

### 2.3. Effect of K^+^ Channels Blockers on Alpinia officinarum Extract-Induced Vasodilation

**Figure 3:**
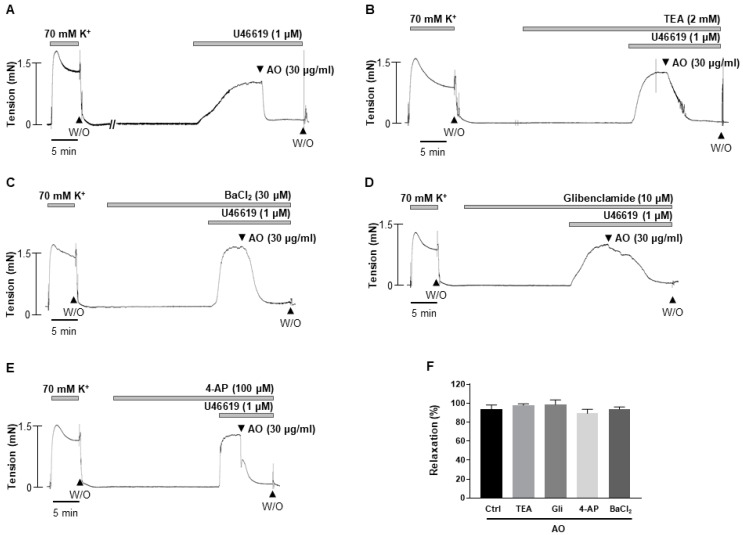
Effect of K^+^ channel blockers on *A. officinarum* extract-induced vasodilation. (**A**), effect of *A. officinarum* extract in the mesenteric artery pre-contracted with U46619 (1 μΜ). (**B**–**E**), effect of *A. officinarum* extract in the presence of TEA (**B**), or BaCl_2_ (**C**), or glibenclamide (**D**), or 4-AP (**E**). (**F**), statistical analysis of the relaxation response of *A. officinarum* extract in the presence of various K^+^ blockers. Relaxation of arteries is expressed as the percentage of the contraction induced by U46619 (1 μΜ). Mean ± SD (n = 5). (AO: *Alpinia officinarum* extract; TEA: tetraethylammonium; Gli: glibenclamide; 4-aminopyridine: 4-AP).

To clarify the underlying mechanisms of the *A. officinarum* extract-induced vasodilation, we examined whether K^+^ channels are involved. The mesenteric arteries were incubated with a non-selective K^+^ channel blocker, tetraethylammonium (TEA, 2 mM, Figure 3B), an inward rectifier K^+^ channel blocker, BaCl_2_ (30 μM, Figure 3C), an ATP-sensitive K^+^ channel blocker, glibenclamide (10 μM, Figure 3D), a voltage-gated potassium channel blocker, and 4-aminopyridine (4-AP, 100 μM, Figure 3E), for 20 min, before being contracted by U46619 (1 μM). We found that all the K^+^ channel blockers administered in this study did not affect the *A. officinarum*-induced relaxation (Figure 3F). These results suggest that K^+^ channel is not involved in *A. officinarum*-induced vascular relaxation.

### 2.4. Effect of A. officinarum Extract on the Extracellular Ca^2+^-Induced Contraction

**Figure 4:**
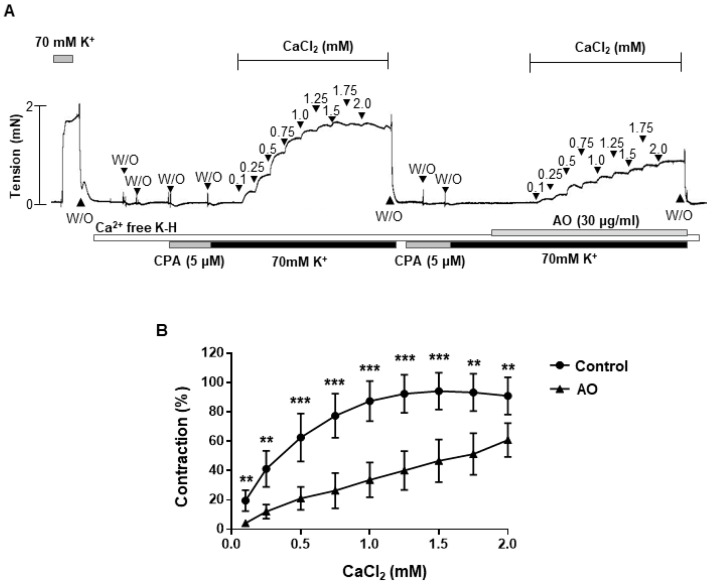
Decrease in Ca^2+^-induced contraction in *A. officinarum* extract-treated mesenteric arteries. (**A**), Representative trace showing the effect of *A. officinarum* in the mesenteric arteries, treated with cumulative addition of CaCl_2_ (0.1–2 mM). (**B**), statistical analysis of contraction induced by CaCl_2_ in the mesenteric arteries, with or without *A. officinarum*. Mean ± SD (n = 7). ** *p* < 0.01, *** *p* < 0.001 (CPA: cyclopiazonic acid; W/O: wash out; AO: *Alpinia officinarum* extract).

To investigate whether *A. officinarum* extract-induced relaxation was related with inhibition of extracellular Ca^2+^ influx, we observed the contractile responses to the cumulative addition of CaCl_2_ (0.1–2 mM) during 70 mM K^+^-induced depolarization, in the presence and absence of *A. officinarum* extract. Pre-treatment of *A. officinarum* extract significantly reduced the contractile responses induced by the cumulative administration of Ca^2+^ (Figure 4), which suggests that extracellular Ca^2+^ influx was inhibited by the treatment of *A. officinarum* extract.

### 2.5. A. officinarum Extract Inhibited Phosphorylation of 20 kDa Myosin Light Chain (MLC_20_) in Vascular Smooth Muscle Cells

**Figure 5:**
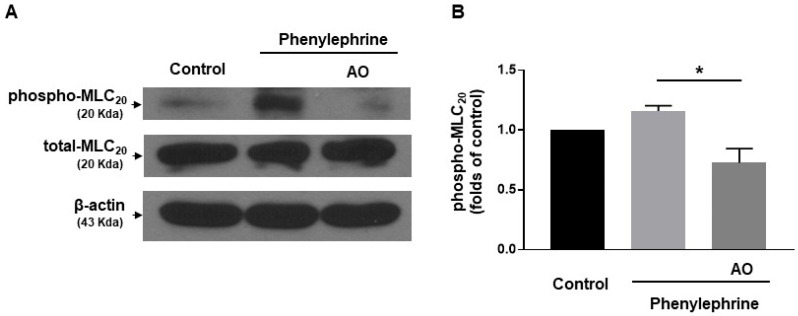
Effect of *A. officinarum* extract on the phosphorylation of 20 kDa myosin light chain (MLC_20_). (**A**), representative Western blot analysis for phosphorylated MLC_20_ (phospho–MLC_20_) and total MLC_20_ (total–MLC_20_) in control VSMCs, VSMCs treated with phenylephrine (5 µΜ), and VSMCs co-treated with phenylephrine (5 µΜ) and *A. officinarum* (30 µg/mL). (**B**). Quantitative data for phosphorylated MLC_20_. * *p* < 0.05 (AO: *Alpinia officinarum* extract).

To investigate whether *A. officinarum* extract-induced relaxation was caused by decreased phosphorylation of MLC_20_, we measured the phosphorylation and expression level of MLC_20_ in vascular smooth muscle cells (VSMCs, Figure 5). The administration of phenylephrine (5 µM) increased phosphorylated MLC_20_ in VSMCs, compared to the vehicle-treated VSMCs. In the presence of *A. officinarum* extract (30 µg/mL), the phosphorylation level of MLC_20_ was significantly reduced (Figure 5B). These data suggested that *A. officinarum* extract inhibited phosphorylation of MLC_20_ in VSMCs.

### 2.6. 1,3,3-. Trimethyl-2-Oxabicyclo [2.2.2]octane (Eucalyptol), an Active Compound of A. officinarum Extract, Induced Concentration-Dependent Vasodilation

**Figure 6:**
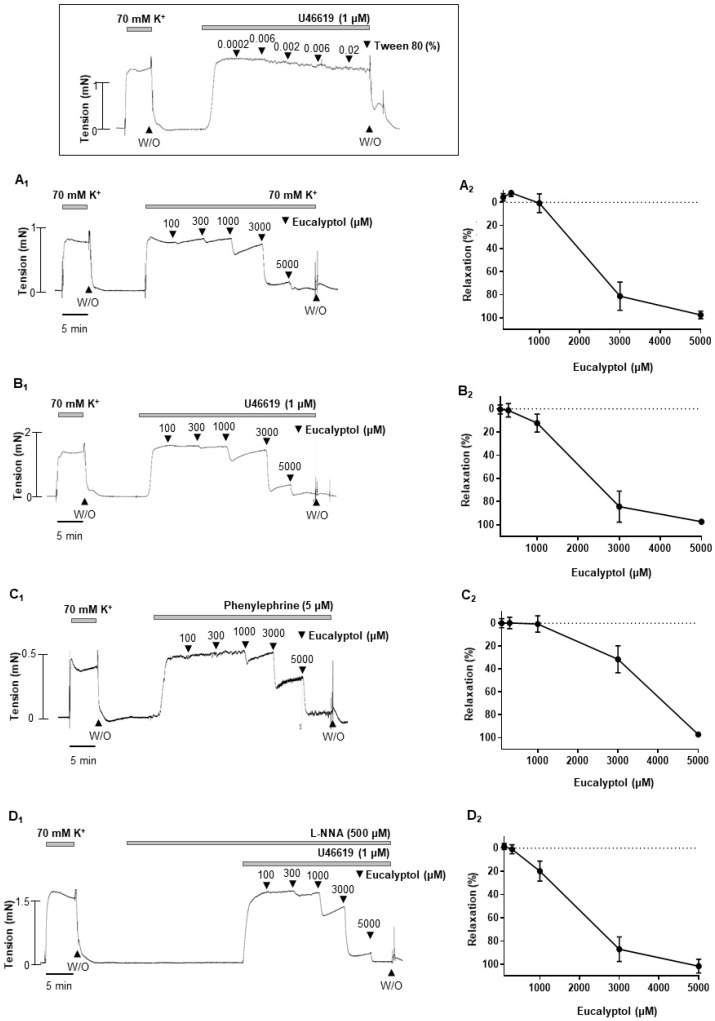
Eucalyptol-induced vasodilation in rat mesenteric resistance arteries. (**A_1_**–**C_1_**), data showing responses to cumulative administration of eucalyptol (100 µM–5 mM) on high-K^+^ (**A_1_**) or U46619 (**B_1_**) or phenylephrine (**C_1_**) -induced contraction. (**A_2_**–**C_2_**), statistical analysis of the relaxation response to eucalyptol. (**D_1_**), effect of L-NNA (500 µM) on the eucalyptol-induced vasodilation. (**D_2_**), statistical analysis of the relaxation response to eucalyptol in the presence of L-NNA. Inset: representative trace showing responses to vehicle, tween 80 (0.0002–0.02%). Mean ± SD (n = 7). (W/O: wash out).

To confirm the vascular effect of *A. officinarum* extract, we investigated the effect of eucalyptol which is an active compound of *A. officinarum*. Eucalyptol (100 µM–5 mM) induced concentration-dependent vascular relaxation in rat mesenteric arteries pre-contracted with high-K^+^ solution (70 mM) or phenylephrine (5 μM) or U-46619 (1 μM) (Figure 6A–C). We also examined whether eNOS is involved in the eucalyptol-induced vasodilation. Incubating the arteries with L-NNA did not alter the relaxation response induced by *A. officinarum* extract (Figure 6D). The vehicle, tween 80 (0.0002–0.02%), had no significant effect on pre-contracted arteries with U-46619. (Figure 6 inset).

## 3. Discussion

The present study demonstrated that *A. officinarum* extract induced a vasodilatory effect in rat mesenteric resistance arteries. We found that *A. officinarum* extract concentration dependently reduced the contraction induced by high-K^+^ (70 mM) or phenylephrine (5 µM) or U46619 (1 µM). In addition, we also found that endothelium- and K^+^ channels are not involved in *A. officinarum* extract-induced vascular relaxation. Furthermore, *A. officinarum* extract inhibited Ca^2+^-induced contraction in the mesenteric arteries. Moreover, phosphorylation of MLC_20_ was decreased by the treatment of *A. officinarum* extract in VSMCs. The active compound of *A. officinarum* extract, eucalyptol, also has a similar relaxation effect to *A. officinarum*.

Previous studies have reported that the total flavonoids from *A. officinarum* induced a protective effect by reducing inflammatory mediators, such as interleukin 1 beta (IL-1β), interleukin 6 (IL-6), tumor necrosis factor alpha (TNF-α), and prostaglandin E2 (PGE2), in in vivo and in vitro models of a gastric ulcer [14]. It has also been reported that *A. officinarum* has an anti-oxidative activity, which is related with superoxide anion scavenging capabilities in vivo and in vitro [13]. Recently, a study reported that an active component of *A. officinarum* has potent anti-proliferative activity in VSMCs, by upregulating cyclin-dependent kinase inhibitor 1B (CDK1B inhibitor, p27^KIP1^) [15]. Although it has been reported that *A. officinarum* has beneficial effects in various diseases and symptoms, no studies have been published on the cardiovascular effect of *A. officinarum*. Thus, this is the first study that shows the vasodilatory effect of *A. officinarum* in rat mesenteric resistance arteries.

Vascular smooth muscle cell relaxation could be directly affected by vasoactive substances or indirectly affected through endothelium. Regardless, this process requires a reduction in intracellular Ca^2+^ concentration, decreased myosin light chain kinase (MLCK) activity, and increased myosin light chain phosphatase (MLCP) activity [16]. A decrease in the phosphorylation of MLC_20_ is, generally, considered the primary mechanism responsible for relaxation in vascular smooth muscle [17].

In the present study, we showed *A. officinarum* extract concentration dependently induced vasodilation in mesenteric arteries pre-contracted with various stimuli such as high-K^+^, phenylephrine, and U46619. To delineate whether endothelium is involved in the *A. officinarum* extract-induced vasodilation, we used not only L-NNA and L-NAME, but also endothelium-denuded mesenteric arteries. The removal of the endothelium and treatment of L-NNA and L-NAME did not affect *A. officinarum* extract-induced vasodilation, suggesting that this effect is independent of endothelium and is not related with nitric oxide (NO) release. These results are not in accordance with the previous report that showed that *A. officinarum* increased endogenous NO generation in the gastric mucosa [18]. However, in that study, *A. officinarum* was treated in a state where the NO level was already reduced by treatment with indomethacin. Since our study investigated the vascular effect of *A. officinarum* in the physiological state, different results could be obtained. Nevertheless, additional studies are needed to delineate the involvement of NO in the effect of *A. officinarum* extract.

Further, we investigated whether *A. officinarum* extract-induced vasodilation involves K^+^ channel activation. The non-selective K^+^ channel inhibitor, TEA, did not alter *A. officinarum*-induced vasodilation. To confirm this result, we incubated arteries with several blockers for different types of K^+^ channels. We found that BaCl_2_, glibenclamide, and 4-AP did not change the effect of *A. officinarum*, which suggests that K^+^ channels including the inward rectifier K^+^ channel, ATP-sensitive K^+^ channel blocker, and voltage-gated potassium channel were not involved in *A. officinarum*-induced vascular relaxation.

To clarify how *A. officinarum* extract induces vasodilation, we examined whether inhibition of Ca^2+^ influx is involved in *A. officinarum*-induced vascular relaxation. We found that treatment of *A. officinarum* extract reduced contractile responses induced by the cumulative administration of Ca^2+^, which suggests that extracellular Ca^2+^ influx was inhibited by the treatment of *A. officinarum* extract. These data are in accordance with the previous study, which showed that the treatment of *A. officinarum* extract decreased Ca^2+^ levels in the uterine smooth muscle tissue of mice [19]. They suggested that *A. officinarum* extract may act on the Ca^2+^ channel to decrease intracellular Ca^2+^ concentration. Furthermore, we found that phosphorylated MLC_20_ is decreased in VSMCs co-treated with *A. officinarum* extract and phenylephrine, compared to VSMCs treated with only phenylephrine. These results suggest that the relaxation of mesenteric artery induced by *A. officinarum* extract involves a decrease in Ca^2+^ influx and, thus, the MLC_20_ phosphorylation.

Although we showed the beneficial effect of *A. officinarum*, further tests were required to confirm whether the single active compound of *A. officinarum* extract also has a vasodilatory effect. Among the compounds identified based on the liquid chromatography–mass spectrometry (LC/MS) and gas chromatography–mass spectrometry (GC/MS) analysis (Supplementary Figures S1 and S2 and Tables S1 and S2), we found that eucalyptol has a similar effect as *A. officinarum* extract. Previous studies also reported that eucalyptol displayed the outstanding bioactivities among the components of *A. officinarum* [20,21]. It is well known that eucalyptol is an essential oil present in various plants [22,23]. This compound is known to be useful for cough, rheumatism, and bronchial asthma [23,24]. In addition, previous studies reported that eucalyptol induced reduction in contractile responses in rat thoracic aorta [25], in rat cardiac muscle [26], and in guinea pig airway smooth muscle [27]. In the present study, we demonstrated that eucalyptol induced vasodilation in a concentration-dependent manner on mesenteric arteries pre-contracted with several stimuli. Our data are in accordance with these previous studies, which show that eucalyptol induces relaxation in multiple types of tissues. However, there is the limitation that eucalyptol is present in the *A. officinarum* extract in very small amounts. Thus, we also tested two active compounds, 4-[(1E)-3-hydroxyprop-1-en-1-yl]-2-methoxyphenol (coniferyl alcohol) and 3-(3,4-dihydroxyphenyl)-7-hydroxy-4H-chromen-4-one, in mesenteric arteries (Supplementary Figure S3). We found that both compounds had a similar effect as eucalyptol. These data from the single active compound support our findings that *A. officinarum* could act as a vasorelaxant.

## 4. Materials and Methods

### 4.1. Animals

All experiments were performed according to the Guide for the Care and Use of Laboratory Animals, published by the US National Institutes of Health (NIH publication no. 85–23, 2011), and were approved by the Ethics Committee and the Institutional Animal Care and Use Committee of Yonsei University, College of Medicine (Approval number: 2020-0148). Animals were housed in individually ventilated caging system cages and in controlled conditions with a light-dark cycle of 12:12 h, 50 ± 10% humidity, and 22 ± 2 °C. Animals had food pellets and water ad libitum.

### 4.2. A. officinarum Extract Preparation

*Alpinia officinarum* was purchased in Gyeong-dong Oriental Medicine Market in Seoul, Korea in 2020 and was authenticated by the Institute of Biotechnology, Korea University, where voucher specimens are maintained. The rhizome of *A. officinarum* (1 kg) was ground into a powder and mixed in ethanol (5 L) by shaking for 24 h at 125 rpm (1.57× *g*). The ethanol extract of *A. officinarum* was filtered through No. 42 filter paper (Whatman International Ltd., Middlesex, England) with five replicates, and evaporated in a rotary evaporator (Eyela, Tokyo, Japan) under reduced pressure at 37 °C. The final extract was stored at −70 °C until use. The *A. officinarum* extract was dissolved in 5% DMSO upon use.

### 4.3. Tissue Preparation

In these experiments, 12-week-old male Sprague Dawley rats were used. Rats were sacrificed with isoflurane (5%), followed by CO_2_ inhalation. To confirm death, we monitored rats for several signs such as no response to toe pinch, no rising and falling of the chest, no palpable heartbeat, and color change opacity in the eyes, as previously described [28]. After we confirmed the death, the heart was removed immediately and the mesenteric artery beds were removed and placed in ice-cold Krebs–Henseleit (K-H) solution (composition in mM: NaCl, 119; CaCl_2_, 2.5; NaHCO_3_, 25; MgSO_4_, 1.2; KH_2_PO_4_, 1.2; KCl, 4.6; and glucose, 11.1). The connective and adipose tissues were removed under an optical microscope (model SZ-40, Olympus, Japan). The second branch of mesenteric arteries (200–250 μm, inner diameter) was isolated and cut into 2–3 mm segments for subsequent analysis.

### 4.4. Isometric Tension Recording

Isometric tension was recorded for testing vascular functionality using a wire myograph system (DMT, Arhaus, Denmark). Briefly, two stainless steel wires (40 µm in diameter) were inserted into the lumen of an artery and mounted according to the methods previously described [29]. After a 30–40 min of an equilibration period in K-H solution bubbled with 5% CO_2_ + 95% O_2_ at 37 °C, arteries were stretched to their optimal lumen diameter for active tension development. Contractility of the arteries was tested by exposure to high-K^+^ (70 mM) solution. The endothelium was mechanically denuded by rubbing the inner surface of an arterial segment with forceps when required. The endothelium removal was confirmed by the absence of relaxation to acetylcholine (Ach, 10 µM) in the U46619 (1 µM) pre-contracted artery. After several wash steps, mesenteric arteries were pre-contracted with high K^+^ (70 mM) or phenylephrine (5 µM) or U46619 (1 µM), and, at the steady maximal contraction, cumulative dose-response curves were obtained for *A. officinarum* extract. To determine the involvement of endothelium, arteries were pre-incubated with L-NNA (500 µM) for 20 min, before being contracted with U46619 (1 μM).

The potential inhibitory effect of *A. officinarum* extract on extracellular Ca^2+^ influx was assessed by incubating endothelium-intact mesenteric arteries with *A. officinarum* extract. We incubated arteries in Ca^2+^-free K-H solution with sarcoplasmic reticulum Ca^2+^-ATPase (SERCA) inhibitor and cyclopiazonic acid (CPA, 5 µM), to deplete the intracellular Ca^2+^ store. Then, the arteries were incubated in a Ca^2+^-free with high-K^+^ solution, followed by a cumulative addition of CaCl_2_ (0.01–2.5 mM) with or without *A. officinarum* extract (30 µg/mL).

### 4.5. Isolation and Culture of VSMCs

Vascular smooth muscle cells were obtained, as previously described [30]. Briefly, after the rats were sacrificed, the aortas were excised, the surrounding fat and connective tissues were removed, and the lumen of the aorta was gently rubbed to remove the endothelium. The aortas were cut into small segments and transferred into a tube containing collagenase (1 mg/mL, Worthington Biomedical Corporation, Lakewood Township, NJ, USA) and elastase (0.5 mg/mL, Calbiochem, San Diego, CA, USA) in Dulbecco’s Modified Eagle Medium (DMEM, Gibco, Waltham, MS, USA) at 37 °C for 30 min. After trituration and centrifugation, the cells were seeded in culture dishes (Corning, New York, NY, USA) and cultivated in DMEM supplemented with 10% FBS, 100 IU/mL penicillin, and 10,000 μg/mL streptomycin (Gibco) at 37 °C, in 5% CO_2_ with a humidified atmosphere. The early passage cells (between two and four) were used.

### 4.6. Western Blot Analysis

The cultured VSMCs were treated with a vehicle (0.05% DMSO) or phenylephrine (5 μM) or phenylephrine (5 μM) with *A. officinarum* extract (30 μg/mL) and, then, homogenized in an ice-cold lysis buffer, as described previously [31]. Western blot analysis was performed for the total MLC_20_ and phosphorylated MLC_20_ (1:1000 dilution; Cell Signaling, Boston, MA, USA). Blots were stripped and then reprobed with the β-actin antibody (1:2000 dilution; Santa Cruz Biotechnology, Santa Cruz, CA, USA) to verify the equal loading between the samples.

### 4.7. Chemicals

The following drugs were used: U-46619 (Tocris Bioscience, Ellisville, MO, USA); acetylcholine (Sigma-Aldrich, St Louis, MO, USA); phenylephrine (Sigma-Aldrich); CPA (Sigma-Aldrich); general laboratory reagents (Sigma-Aldrich); eucalyptol (Sigma-Aldrich); 4-[(1E)-3-hydroxyprop-1-en-1-yl]-2-methoxyphenol (Cayman, Ann Arbor, MI, USA); and 3-(3,4-dihydroxyphenyl)-7-hydroxy-4H-chromen-4-one (Biosynth Carbosynth, Compton, Newbury, UK).

### 4.8. Statistical Analysis

The data were expressed as the mean ± SD and were analyzed by one-way or two-way ANOVA with Tukey’s post hoc test. Values of *p* < 0.05 were considered significant. GraphPad Prism (Version 7, GraphPad software, La Jolla, CA, USA) was used for the statistical analysis.

## 5. Conclusions

In the present study, we showed that *A. officinarum* extract induced concentration-dependent vasodilation in the rat mesenteric resistance arteries. The vasodilatory effect of *A. officinarum* extract was endothelium independent. The inhibition of extracellular Ca^2+^ influx was related with *A. officinarum* extract-induced vasodilation, which was associated with a decrease in MLC_20_ phosphorylation. The single active compound of *A. officinarum*extract, eucalyptol, also induced vasodilation in rat mesenteric resistance arteries. These results suggest that the administration of *A. officinarum* extract could exert beneficial effects to treat high blood pressure.

## Data Availability

Data are available upon appropriate requests.

## Supplementary Materials

The following supporting information can be downloaded at: https://www.mdpi.com/article/10.3390/molecules27092711/s1, Figure S1: Representative liquid chromatography–mass spectrometry (LC/MS) of *A. officinarum* extract; Figure S2: Gas chromatogram of the compounds in *Alpinia officinarum* extract; Figure S3: Effect of single active compound of Alpinia officinarum on U46619-induced contraction in rat mesenteric arteries. A. 4-[(1E)-3-hydroxyprop-1-en-1-yl]-2-methoxyphenol (coniferyl alcohol, 50–300 μM) B. 3-(3,4-dihydroxyphenyl)-7-hydroxy-4H-chromen-4-one (3’, 4′, 7-THIF, 50–300 μM) Inset. Representative trace showing responses to vehicle, DMSO (0.003–0.3%). (*W*/*O*: wash out); Table S1: LC/MS profile of *Alpinia officinarum* extract; Table S2: Bioactive compounds detected in *Alpinia officinarum* extract.

## Sample Availability

Samples of the compounds are not available from the authors.
