# Supplementary material for: Vasodilatory Effect of Alpinia officinarum Extract in Rat Mesenteric Arteries
Source: Molecules. 2022 Apr 22;27(9):2711. doi: 10.3390/molecules27092711 (PMC9104054; doi:10.3390/molecules27092711)
Supplement: Supplementary file 1 [file molecules-27-02711-s001.zip › molecules-1696133-supplementary.pdf]

Supplementary materials

1. Liquid Chromatography–Mass spectrometry (LC/MS) of the Compounds in *Alpinia officinarum* Extract

The liquid chromatography–mass spectrometry (LC/MS) of the extract of *A. officinarum* is shown in Figure 1. LC-MS analysis of *A. officinarum* extract had detected several peaks with the retention time 0.623, 0.639, 0.641, 0.641, 0.651, 0.660, 0.673, 6.128, 6.489, and 6.901 minutes (Supplementary figure 1). Then each peak was fragmented, resulting 10 fragmentation spectrum with candidate mass 294, 342, 144, 179, 117, 103, 265, 324, 328, and 284 (Supplementary figure 1 and Supplementary table 1)

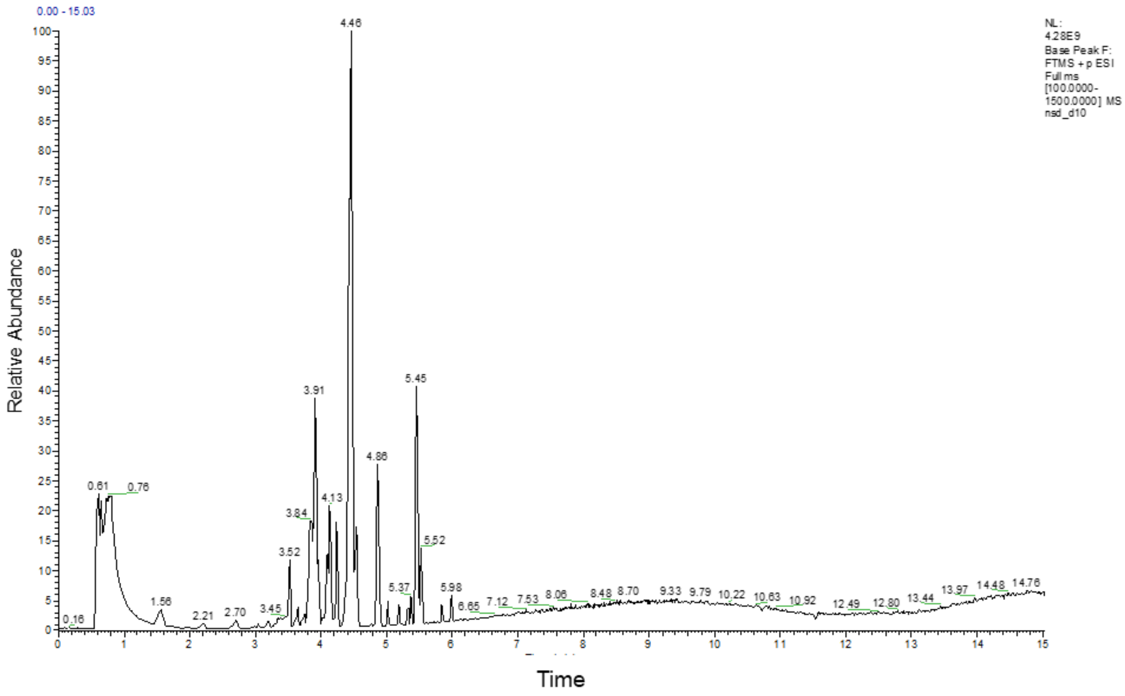

Supplementary Figure S1. Representative liquid chromatography–mass spectrometry (LC/MS) of *A. officinarum* extract.

No.	Formula	Candidate mass	RT [min]	Area (Max.)
1	$C_{12}H_{22}O_{11}$	342.11563	0.639	9316875.179
2	$C_{21}H_{28}N_2O$	324.21929	6.128	7395611.184
3	$C_{20}H_{24}O_4$	328.16682	6.489	2361769.912
4	$C_5H_{11}NO_2$	117.07903	0.651	2132782.077
5	$C_5H_{13}NO$	103.09992	0.660	1744328.472
6	$C_{10}H_{19}NO_7$	265.11581	0.673	1186559.267
7	$C_{16}H_{12}O_5$	284.06771	6.901	617141.770
8	$C_6H_8O_4$	144.04211	0.641	595519.273
9	$C_6H_{13}NO_5$	179.07915	0.641	538513.823
10	$C_{10}H_{18}N_2O_8$	294.10585	0.623	531614.988

Supplementary Table S1. LC/MS profile of *Alpinia officinarum* extract.

2. Gas Chromatograms of the Compounds in *Alpinia officinarum* Extract

The gas chromatogram of the compounds identified in the extract of *A. officinarum* is shown in Supplementary figure 2. The identities of eight compounds were determined along with their retention time (Supplementary table 2). The compounds identified based on the gas chromatography–mass spectrometry (GC/MS) analysis include 1,3,3-trimethyl-2-oxabicyclo[2.2.2]octane, 3-phenylpropanal, 2-(4-methylcyclohex-3-en-1-yl)propan-2-ol, 4-phenylbutan-2-one, 4-[(1E)-3-hydroxyprop-1-en-1-yl]-2-methoxyphenol,3-anilino-2-cyclobuten-1-one, (2S)-2-azaniumyl-3-(4-hydroxy-3-methoxyphenyl)-propanoate, 3-(3,4-dihydroxyphenyl)-7-hydroxy-4H-chromen-4-one. (Supplementary table 2).

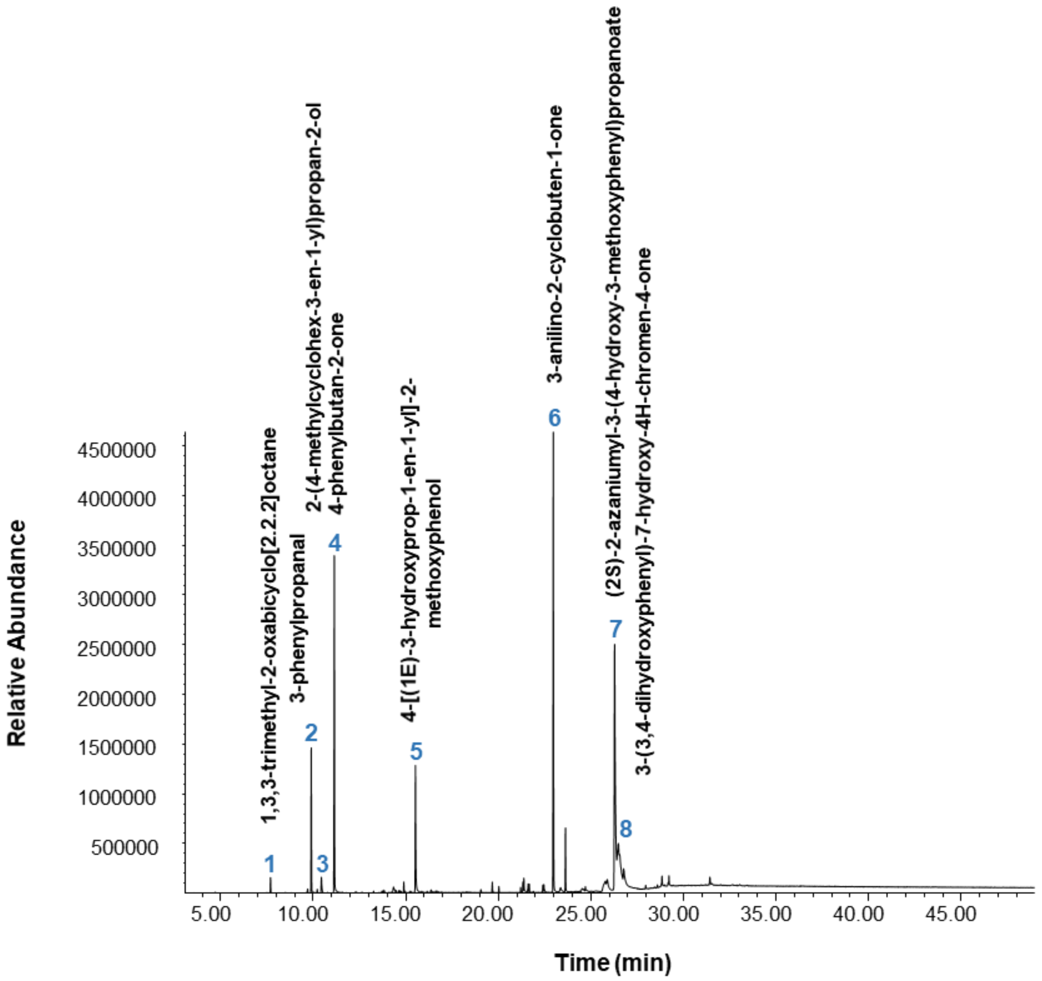

Supplementary Figure S2. Gas chromatogram of the compounds in *Alpinia officinarum* extract.

No.	Compounds	RT (min)	Total (%)
1	1,3,3-trimethyl-2-oxabicyclo[2.2.2]octane	7.70	0.64
2	3-phenylpropanal	9.90	6.03
3	2-(4-methylcyclohex-3-en-1-yl)propan-2-ol	10.45	1.04
4	4-phenylbutan-2-one	11.15	14.11
5	4-[(1E)-3-hydroxyprop-1-en-1-yl]-2-methoxyphenol	15.53	6.85
6	3-anilino-2-cyclobuten-1-one	22.97	19.58
7	(2S)-2-azaniumyl-3-(4-hydroxy-3-methoxyphenyl)propanoate	26.28	24.24
8	3-(3,4-dihydroxyphenyl)-7-hydroxy-4*H*-chromen-4-one	26.49	7.60

Supplementary Table S2. Bioactive compounds detected in *Alpinia officinarum* extract.

3. Effect of Single active Compound of *Alpinia officinarum* on U46619-induced Contraction

To confirm the vascular effect of *A. officinarum* extract, we investigated the effects of active compounds of *A. officinarum*. 4-[(1E)-3-hydroxyprop-1-en-1-yl]-2-methoxyphenol (coniferyl alcohol, 50–300 μM) and 3-(3,4-dihydroxyphenyl)-7-hydroxy-4H-chromen-4-one (3', 4’, 7-THIF, 50–300 μM) induced concentration-dependent vascular relaxation in rat mesenteric arteries (Supplementary figure 3).

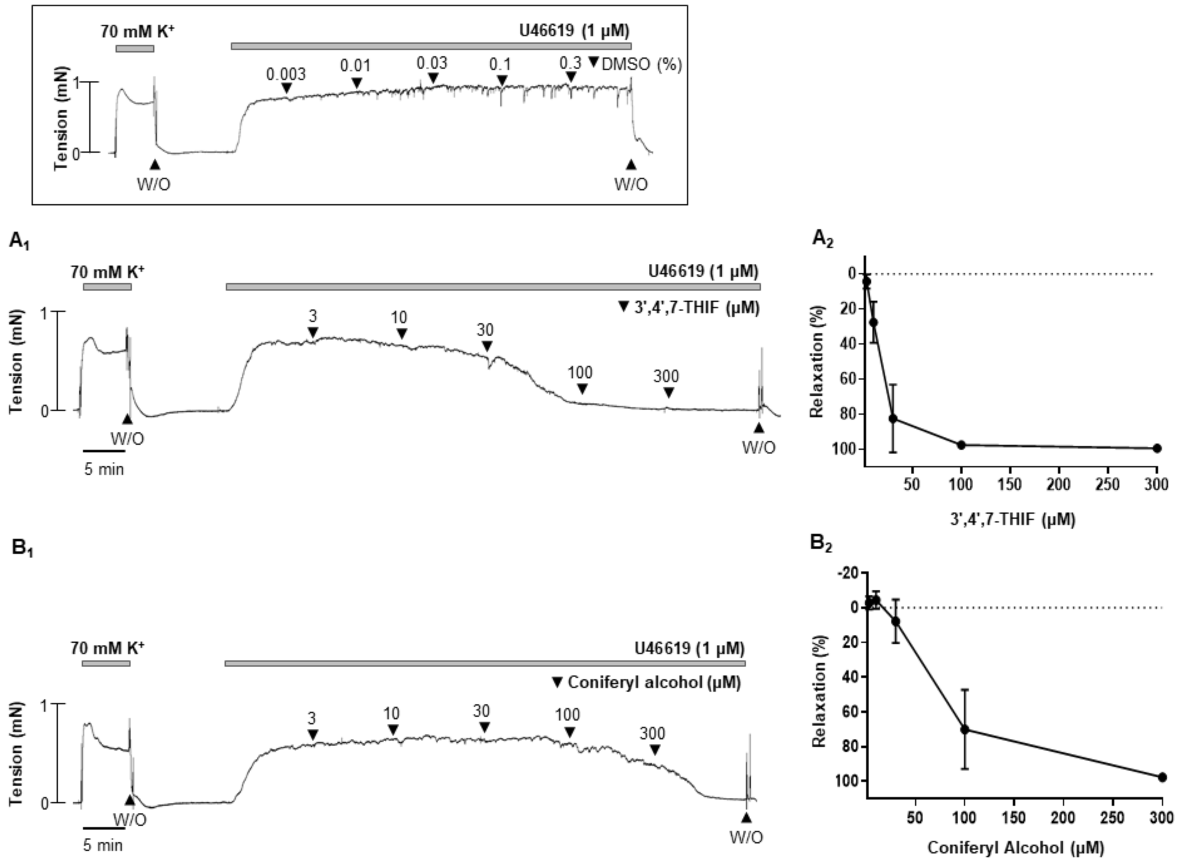

Supplementary Figure S3. Effect of single active compound of *Alpinia officinarum* on U46619-induced contraction in rat mesenteric arteries. A. 4-[(1E)-3-hydroxyprop-1-en-1-yl]-2-methoxyphenol (coniferyl alcohol, 50–300 μM) B. 3-(3,4-dihydroxyphenyl)-7-hydroxy-4H-chromen-4-one (3', 4’, 7-THIF, 50–300 μM) Inset. Representative trace showing responses to vehicle, DMSO (0.003–0.3%). (W/O: wash out)

Method

The Liquid Chromatography–Mass Spectrometry (LC/MS) Analysis

The extract of *Alpinia officinarum* was analyzed using nano-ESI with a ThermoFisher LTQ Orbitrap XL LC/MS (Waltham, MA, USA). 30 µl of sample was loaded onto a 5 mm, 75 µm ID C12 vented column (Jupiter Proteo, Phenomenex, Torrance, CA, USA) at a flow rate of 10 µl/min. A 30 min gradient elution was conducted over a 15 cm, 75 µm ID C12 column at 300 nl/min. The Orbitrap MS scan was performed at 60,000 FWHM resolutions.

Gas Chromatography–Mass Spectrometry (GC/MS) Analysis

GC/MS analysis was performed by an Agilent 7890B gas chromatograph, equipped with a 5977A mass selective detector quadrupole mass spectrometer system (Palo Alto, CA, USA). The DB-5 MS capillary column (30 m × 0.25 mm i.d., 0.25 µm film thickness, 5% diphenyl-95% dimethylsiloxane phase) was obtained from J&W Scientific (Folsom, CA, USA). The GC oven temperature was maintained at 60°C for 3 min, and then ramped to 320°C at 10°C per min. The sample was injected in the split mode, at a splitting ratio of 1:30. The temperatures of the GC injection port and MS interface were set at 300°C. The mass selective detector was run in the electron impact (EI) mode, with an electron energy at 70 eV. The mass spectrometer was operated in the full scan mode between 40 and 600 amu. For the identification of the compounds, EI mass spectral library search (Wiley registry 7n edition, Wiley Science Solutions, Hoboken, NJ, USA) was used.
